# How the Sarah Everard Case has Changed Public Perceptions of Police Officers

**DOI:** 10.1177/10778012251366237

**Published:** 2025-08-11

**Authors:** Summer Rita Josephine Herbert, Maria Ioannou, Calli Tzani, Rachel Fletcher, Thomas James Vaughan Williams

**Affiliations:** 1 Department of Psychology, School of Human and Health Sciences, 14270 University of Huddersfield, Huddersfield, UK

**Keywords:** police distrust, social media, public perceptions, fear, anxiety

## Abstract

News can have profound effects on public perceptions of the police. The public's views of the police following the widespread case of Sarah Everard have yet to be examined, despite it's link to violence against women and girls. This research explored the impact of this case on public perceptions of the police in the period afterwards. An analysis of 107 social media posts uncovered seven themes showing this case caused extreme fear and anxiety. While some viewed the incident as a “bad apple,” most tweets showed distrust toward the police force as a whole, raising repercussions for police–public relationships and reporting decisions.

## Introduction

Citizens’ trust in and fear of the police has arisen as an important topic of concern in the past decade (De Angelis & Wolf, 2016). With a newfound age of social media, everything nowadays is given the opportunity to be documented, reported on, and discussed online. For instance, a string of events showing police brutality, bias, and racism perpetrated by law enforcement were reported during the Height of COVID-19 in 2020 (Lai & Zhao, 2010; Nix & Wolfe, 2017). The killings of Breonna Taylor, a 26-year-old EMT, and George Floyd, by serving officers in 2020, resulted in a national outrage (The New York Times, 2022). The effects of these publicized incidents on the public’s fear of the police were severe; college students, particularly those who were racially underrepresented like the victims, were experiencing fear for their own safety and for future police encounters (Campbell & Valera, 2020). One participant shared: “it also adds to my already high levels of distrust for the police and our criminal justice system” (Campbell & Valera, 2020, p. 663). Recent studies by Murray and McGovern (2014) and Colbran (2018) also suggest that social media can play a huge role in influencing the public's perceptions of policing, often increasing distrust through the rapid spread of mediated stories.

Research proposes several factors affecting citizens’ trust toward the police. As shown in the aforementioned research, Weitzer and Tuch (2005) describe that those who have been wronged or have witnessed bad behavior toward the public, or those who look like them, are likely to develop lower overall confidence levels in how the police operate and conduct their daily work. Individuals who have had negative experiences with the police may have their judgments and opinions skewed by their experiences (Machura et al., 2014, 2019). As suggested by Willis and Todorov (2006), first impressions are key to a good relationship and are an important way in which perceptions are formed. It will just take as little as 100 ms to develop an opinion on someone, which is why it is extremely important that the police understand public perceptions of themselves and how officers’ behaviors with the public can affect how the public view other officers in the future (Awan et al., 2017). The “bad apple” phenomenon suggests that individuals who express negative behaviors and emotions toward others can dramatically lower others’ confidence levels in a wider group (Felps et al., 2006). As such, one bad experience can damage the reputation of those whom one has not even encountered, yet they automatically fear. This supports Mawby's (2010) analysis of police “image work,” where the public's trust can quickly break down as they hear of policing failures.

Investigating the causes of police distrust in the United Kingdom, Machura et al. (2019) found that among factors such as politics, previous bad experiences, culture, and the news, the news had a significant influence over how positively the police are viewed. Research conducted by ABC News (1996) found that 75% of the public form their opinions on crime and offending through what they hear and read in the news. Similarly, Doyle (2003, pp. 14–15) suggests that watching the news is like a “shared arena,” meaning that the public does not decide what they would like to hear; instead, they have no choice in what they view. Eventually, the public will perceive these media reports as their reality, and this will influence how they view their nation's crime policies (Garland, 2000). Smolej (2011) argues that consistently reporting crimes in the media causes fear among the public. This is supported by Altheide (2002, 2006), who suggests that media reporting is highly influential in causing nationwide fear and distress to the public. As Ellis (2021) suggests, the media can play a vital role in how people view the police, and how repeated exposure to negative news coverage can lower the public’s trust and confidence. Similarly, Murray and McGovern (2014) found that the way the media presents stories to the public can shape how they view and understand policing work.

Similar to Campbell and Valera (2020), studies by Lawrence (2000) and Weitzer and Tuch (2004) investigated heavily publicized police crimes in the media and how this influenced public perceptions of officers. Both found similar results, which suggested that those who consistently watch and view these types of crime reports in the news will be more aware of these types of incidents, and subsequently, this would negatively impact their attitudes toward the police. Cultivation theory is concerned with the media’s effect on public perceptions, and within the last 20 years, this has been considered in regard to news media. Romer et al. (2003) argue that although the media reflects events in the world which are important, the structure of the news cultivates unrealistic fears in the public which are independent of real-life crime rates. In other words, uncommon incidents can cause disproportional fear and anxiety among a population. “Mean world syndrome” is described as an outcome of cultivation, with research finding that heavy media consumers perceive the world with more menace than in reality and believe that others cannot be trusted. Similarly, Intravia et al. (2018) found that reading news online negatively influences attitudes toward the police, and Dowler and Zawilski (2007) found that viewing negative news about the police through media actively influences and shapes whether public perceptions of the police will be positive or negative. Recent work by Simpson et al. (2024) explores how such crime news can have a “haunting” effect, which can leave a lasting emotional effect and impact on the public's feeling of safety.

With this in mind, it is unsurprising that fear is increasing in the British public when it has been reported that in 2020, over 211 police officers and PCSOs were employed with criminal convictions (Mercer, 2020), and some forces still refuse to disclose accurate information. Moreover, in March 2022, a total of 812 officers in England and Wales were dismissed from duty and added to the “barred list” from December 2017 to March 2021 (College of Policing, 2022). Following the highly publicized abduction, rape, and murder of Sarah Everard in March 2020 by a serving metropolitan police officer, a review of “thousands” of messages exchanged by metropolitan officers described much of the content as misogynistic, highly sexualized, discriminatory, and referring to violence (Brunt, 2022). Sarah Everard was a 33-year-old female, who, while walking home from a friend's house on the night of March 3rd, 2020, was reported missing while on the phone to her partner (Morton, 2021). The nation was shocked when it was later revealed that Wayne Couzens, the aforementioned metropolitan police officer, had hired a car which looked similar to a police car and used his police badge to falsely arrest Sarah for “breaking lockdown restrictions,” and subsequently abducted, raped, and murdered her (Dodd & Siddique, 2021). Couzens was only fired from the police force after pleading guilty to his crimes, while also having allegations of indecent exposure just one month prior to Sarah's death (Thompson et al., 2021). Linnemann (2022) suggests that such high-profile incidents contribute to the “horror of police”; those among marginalized communities may view the police as not just flawed, but dangerous too.

Research is limited on public perceptions of police officers after a publicized tragedy such as Sarah Everard’s, yet we understand that heavily publicized police crimes have previously influenced public perceptions of officers (Lawrence, 2000; Weitzer & Tuch, 2004). Resulting from Sarah's case, several worrisome statistics have been reported due to forced transparency with statistics and figures surrounding these issues. One year before Sarah Everard's murder by an active police officer, there were a total of 28,223 complaints against police officers recorded for the year 2019/2020 (IOPC, 2020), with a total of 54,015 allegations nationwide. A total of 2,250 of those were investigated, and a large 17,608 of those complaints were not investigated further. 80% of those complaints were against White officers, and 69% were male officers (IOPC, 2020). 85% of the complaints were against police officers. These statistics are highly concerning, yet the impact which publicized content of this sort could have on public perceptions is yet to be explored. Theories such as “signal crimes,” as explained by Innes (2004), show how certain incidents like Everard's murder may serve as powerful and symbolic events that can heighten community trust and fear. Feminist research into fear of crime, like that of Stanko (1995), Rader (2004), and Fanghanel (2020), shows that experiences of fearing crime can affect people differently, disproportionately affecting women and marginalized groups.

### The Current Study

It is crucial that women and girls are able to feel safe in the outside world and feel as if police officers are there to protect them, not cause them harm. The aim of the current research is to qualitatively explore the impact which the heavily publicized case of Sarah Everard had on public perceptions of police officers, to raise awareness of the consequences of this. Of the previous studies concerned with public perceptions of the police, limited research attends to this context in Britain, or after reports whereby a police officer committed an act of violence against women or girls, most notably shown in the case of Sarah Everard.

## Method

### Data

A total sample of 107 “tweets” (i.e., posts) was collected from 107 different social media accounts via the online media platform “Twitter.” These tweets comprised a total of 1,156 words. The present study selected tweets written between March 3rd, 2021 and July 11th, 2021, best demonstrating the state of public perceptions in the months after Metropolitan Police Officer Wayne Couzens was arrested and pleaded guilty to Sarah Everard’s murder.

### Procedure

Twitter was the only social media platform used in this research. A professional account was created to manage and maintain all Twitter posts within the criteria. Various search words were used to generate specifications of the study topic on the explore page; “Sarah Everard,” “Sarah Everard murder,” and “Wayne Couzens” were searched while filtering dates between March 3rd and July 11th, 2021. This led to various different hashtags, for example, #shewaswalkinghome, #justiceforsarah, #reclaimthestreets, #RIPSarahEverard, and #toomanywomen. Other search words and phrases were also used to generate tweets for analysis: police failures in the United Kingdom, distrust in the British police, untrustworthy, conduct, police crimes, scared, police anxiety, police fear, while also filtering for Sarah Everard or Wayne Couzens. During the selection process, it was ensured that there was one account per tweet; therefore, each opinion came from different persons. There would be no repeat accounts in the analysis.

### Data Analysis

The Twitter posts were analyzed using Braun and Clarke's (2006) thematic analysis, following the six-step guide:

1. Familiarization with the data—The tweets were read and re-read throughout this phase analysis to establish enough familiarity with the data and to detect potential codes (characteristics relevant to the present study's research question). Notes were made to summarize the data and allude to parts which revealed the impact of Sarah Everard’s case on the public's perceptions of the police.
2. Generating initial codes—Initial codes and their related tweet extracts were combined into another document and sorted into meaningful groups. These were recorded on separate transcripts as well as in a master document summarizing emergent codes and interpretations seen across tweets.
3. Searching for themes—The codes were organized into possible themes (i.e., patterns of meaning which recur within the codes). Themes were identified if they were repeatedly observed across the data.
4. Reviewing themes—The themes were repeatedly examined and refined to ensure there was sufficient evidence across the tweets to support each theme (it appeared significant in the data), and that the data provided meaning to the research question.
5. Defining and naming themes—The themes were clearly defined and named in accordance with the meaning they provide to the research question.
6. Producing the report.

The analysis followed an essentialist inductive approach, meaning the themes were data-driven and free from analytic preconceptions. This allows for a more subjective analysis (Braun & Clarke, 2006).

## Results

A thematic analysis was conducted on 107 tweets (i.e., Twitter posts) each made by different users and accounts. This addresses the research aim of the current study—to examine the impact that Sarah Everard’s case had on public perceptions of the police. The analysis identified seven themes: “Who is there to protect us?”; “Emotional Outcome”; “Distrust in the police” with one subtheme “Male distrust”; “Misogyny in the forces”; “Women do not feel safe”; “The police do not care about us”; and “Bad apples among every bunch”.

### Superordinate Theme 1: Emotional Outcomes

It was apparent from the findings that there were two main themes, which were consistently identified throughout the analysis of the tweets. “Emotional Outcomes” was the largest of the seven themes. Ninety percent of all tweets analyzed that viewed police officers negatively linked to the Sarah Everard case. Forty-two of the analyzed tweets identified emotional outcomes, relating to fear and to their perceptions of police officers. Some examples include: “Shivers of fear,” “I live in fear,” “We are always in fear,” and “Our daily lives should not be lived in fear.”

A key finding from this analysis was that fear was experienced by members of the public regarding police forces and officers. They describe living in fear of officers, and contact with an officer would cause them great fear and anxiety. They suggest that after hearing about a metropolitan police officer being arrested for a crime such as murder, they will never be able to confide in them again. This affects them not only emotionally and psychologically, but also physically, as they feel that nobody is there to protect them. One tweet said, “because of the fear, I will not receive back up from the police if I am in danger.”

Similar to fear, several tweets showed the anxiety caused by the Sarah Everard case, relating to how a police officer could commit such a crime, for instance:

“Intense anxiety,” “I’m feeling really anxious,” “The Sarah Everard case makes me feel so scared, anxious and sad,” “Operate in a permanent state of anxiety,” “A met police officer being arrested only heightens the anxiety.”

The case appears to affect some users’ anxiety levels on a permanent basis, and therefore, could be hesitant to ask a person of authority for help due to the fear and anxiety that what happened to Sarah would happen to them. One tweet said, “every woman's fear has now come to life for Sarah Everard,” suggesting that women have potentially been fearing these types of attacks. Women have been verbally attacked by the police since the 1970s, when police failed to catch Peter Sutcliffe for the murders in West Yorkshire and Manchester. The police often blamed the victims and branded them as dispensable, and women were blamed for going out alone (Pidd & Topping, 2020). The Byford report found systematic failures of the police in their duties to keep the public protected (Byford, 1981), similar to how the public is feeling after the Sarah Everard case.

Participants wrote how “my heart breaks” and “I am heartbroken” at what has happened to Sarah, “it is enraging,” “I hate the police,” and “I can’t find the words to express how flaming angry I am,” are what the British public posted. They are angry and hurt with the police, and do not have faith in them, “the public should not have to live in constant fear.”

One user described: “This is trauma.” This user expresses that this case is deeply distressing for themselves and/or others. Trauma is defined as any stressful, frightening, or distressing event that can cause emotional, psychological, or physical harm (Horowitz, 1989). This could suggest that with the high media presence of the murder case, and the fact that the offender is a police officer, it could be highly distressing for many other people who may have been victims of similar crimes in the past. Women have been subject to years of fear and abuse because of criminals and offenders; within just 28 weeks after Sarah Everard’s murder, 81 women had been murdered by male suspects (Smith, 2021). Femicide statistics are high, and have been over a 10-year period (Femicide Census, 2018, 2020), and a police officer committing this type of crime is likely to add to the hurt, anger, and fear experienced by the public.

Through use of the words “walk in peace” and “always,” it is suggested that this fear occurs throughout women's daily activities: “Want the right to walk in peace without fear of attack,” “Women should not have to fear for their safety,” “It's about the fear females always have, and how we can’t even trust male officers.”

The public, especially females, worry about walking home at night, or being followed. Women are often told to stick to a curfew when there is a predator about, for instance, the police implemented a curfew for women to stay inside after dark to protect themselves against the Yorkshire Ripper, opposed to a curfew for males who pose greater risk (Bindel, 2006). Since the new age of social media, and crimes being reported more often in the news, it is difficult to escape worrying details of many homicide cases which have happened across the United Kingdom in the last 10 years. Murders, rapes, sexual assaults, harassment, and other crimes are reported daily in the news, many against women. The latest Femicide Census (2020) report shows that 38% of victims were murdered due to a sharp instrument/knife, 4% of cases involve sexual violence, and 6% of the perpetrators had previous abuse of stalking and harassment. Research shows that one woman is killed every 3 days in the United Kingdom (Femicide Census, 2018); women already feel fear and anxiety on a general scale in society in their daily lives, and there is an added concern now that the police force will not always protect them. The tweets suggest that there was already fear and anxiety surrounding the police for some of the population; however, Sarah Everard's murder appears to have heightened public emotions toward the police.

### Superordinate Theme 2: Distrust in the Police

The second largest theme was “Distrust in the Police.” Thirty-seven of the tweets analyzed showed levels of distrust for police officers and the police force, as well as men in general. The latter point led to the identification of the subtheme “Male distrust.” This subtheme is small, but clearly significant:

“Can’t trust any man now,” “This is why women don’t trust men,” “All men are trash,” “The problem, men.”

Importantly, not all males, and not all male officers, are criminals or offenders. However, the British public writes about being afraid and anxious over not being able to trust anybody, a police officer or a man, because they do not know which men or officers are good and which ones are bad and therefore are frightened of them all. The Sarah Everard murder has reaffirmed their distrust in men, and it appears that trust will be impacted by this case for the foreseeable.

“Our lives will forever be changed by Sarah Everard,” “Why don’t women trust the police? Sarah Everard, that's why!,” “First thing that comes to mind when we have encounters with the police.”

Sarah's case has changed the public perceptions of police officers of the many individuals who actively use social media. Many of the tweets suggested that her murder is the reason why the level of police distrust has increased significantly in the past year and a half. Interestingly, this also changes how individuals think they will interact with the police in the future. Because of Sarah, the tweets suggested that they will “think twice when approached by a plain-clothed or lone officer,” and many stated that they would ensure they knew their rights beforehand. For years, many will remember this case, and this will affect how they view and judge various situations, make individuals view law enforcement negatively, and place distrust in authority figures. This is described by several tweets in relation to the Sarah Everard case:

“Destroyed public trust,” “We can’t even trust the police,” “I now have no trust in the police,” “They’ve only fomented distrust and division,” “Trust in the police is at an all-time low.”

If we cannot trust the police, who can we trust? This is one of many questions the public asked themselves. Some stated that they cannot trust those whose role is to protect communities and bring justice, so who could they turn to if they needed help? They do not trust police officers as individual people because of the Sarah Everard case, therefore, they “actively distrust them all.”

### Superordinate Theme 3: Women Do Not Feel Safe

“Women do not feel safe” was also a significant theme derived from the analysis. The theme alludes to the finding that many tweets showed that women do not feel safe around the police, or that they do not feel the police's intention is to do so:

“Police haven’t made strides in keeping us safe,” “I will never feel safe around a police officer again,” “Women are not safe on the streets,” “I very rarely feel safe,” “Feel unsafe with the British Police Force.”

There were many accounts of individuals stating that they felt unsafe regarding the police force and officers, in particular, one user tweeted: “Sarah did everything to stay safe.” This would suggest that, because Sarah did everything in her power to stay safe, and she was still murdered, what more do women have to do to stay safe on the streets of Britain nowadays? Women can do everything in their power to stay safe, yet they will never truly be safe; this is why there is so much anxiety, fear, and distrust among the British public and authority figures.

### Superordinate Theme 4: Misogyny in the Forces

The fourth theme identified is “Misogyny in the forces,” which identifies people's sexist worries within the police force:

“Sexist,” “Misogynistic,” “With power, comes sadism.”

The analysis showed the public already held views that sexism and misogynism existed in the police force before Sarah's murderer became public knowledge. However, this case adds to the already existing sexist views and perceptions people already had toward the police force:

The same misogynistic mindset the Yorkshire Ripper's 22 victims faced 40 years ago.

This statement suggests that the British public feel that no progress has been made to protect women and make them feel safer on the streets of the United Kingdom in 40 years.

### Superordinate Theme 5: Who Is There to Protect Us?

The fifth theme identified is “Who is there to protect us?.” Because of Sarah's murder, the analysis shows that the public is constantly questioning who is supposed to help them in a time of need.

How are we to know whom to trust amongst the police force now?

This is the most compelling statement; if the British public cannot trust police officers, who can they trust? This makes them rethink their entire perceptions of officers. As they do not know which officers are good and which ones are bad, they will automatically distrust all police officers:

“Who are supposed to protect women?,” “How does society expect women to feel safe?,” “How are women ever meant to feel safe?.”

There is an overall societal fear among women, because of this case; they feel like they are never going to feel safe around a police officer again, on the streets or in their communities. They feel on guard, while also feeling like no one is there to protect them if they need help:

Those who are meant to protect us are the worst offenders in their ranks.

The above statement suggests that they do not trust or feel safe around the police force to trust and protect them, as they are also capable of committing serious crimes.

### Superordinate Theme 6: The Police Do Not Care About Us

The sixth theme identified is “The police do not care about us,” which includes statements that suggest that the police do not care for the British public:

“They can harm and abuse you in a time of need,” “The police do not act in our best interests,” “The police don’t want to protect us,” “They don’t want to change.”

The British public feel that it is “one rule for them and one rule for us.” How was it possible for the police to not notice such an offender among them? They act for themselves and not for the public. This suggests that the public feels that, despite Sarah's murder, the police may not act in the interest of public. Since Sarah's murder and the huge media recognition that came with it, the police have come under enormous scrutiny. In turn, public perceptions have become increasingly negative toward officers.

### Superordinate Theme 7: Bad Apples in Every Bunch

The final theme identified is “Bad apples in every bunch,” which is a positive theme compared to the previous six themes identified. Ten tweets perceived police in a more positive manner following the Sarah Everard murder, supporting the notion that Wayne Couzens was simply a bad apple:

“Did we distrust medics and nurses after Shipman and allot?,” “Wasn’t a sudden national distrust of GPs after Shipman,” “Harold shipman did not stop the public from using the NHS.”

These statements suggest that just because of this case, not all police officers are to be feared. The British public did not stop using doctors after the Shipman revelations, so why should we treat the police any different?

“Bad apples everywhere,” “I don’t think we should distrust the police as a direct result of Sara Everard,” “It doesn’t automatically make me distrust the police,” “The actions of one man cannot be attributed to the whole of our police service,” “I still trust the police,” “A bad apple.”

These tweets argue that it would be unfair to tarnish the reputation of the police force because of the actions of one officer. It is unfair to the good officers, and those are the ones who suffer, there will always be bad people in all walks of life.

Abusers and violent people come from all walks of life.

From this statement, it is suggested that anybody can commit a crime, no matter what uniform they wear. Therefore, it is unfair to judge and distrust the police at the hands of what one person did. Their direct perceptions of the police have not changed, as they believe that the actions of one man should not generalize an entire population of officers.

## Discussion

The purpose of the current research was to understand how the case of Sarah Everard impacted public perceptions of police officers, after the circumstances surrounding the case were published. Seven themes were identified following the analysis of 107 tweets. From this, one of the main findings was fear and anxiety which arose surrounding police officers and encounters with them. Users shared emotions, such as feeling scared, anxious, and overcome with fear at the thought of needing a police officer in the future. This was particularly the case for women, with some stating that they do not trust any police officer or male officer/person now, because of what happened to Sarah.

This could be explained by Poteyeva and Sun (2009), who conducted a study which assessed gender differences in police officer attitudes. They found that female officers showed greater levels of compassion and empathy and would conduct their jobs in a manner which was more professional than their male colleagues. This ties in with Bradford's (2011) and Byrne and Jarman's (2011) suggestion that previous bad experiences can shape how individuals perceive the police. Mishler and Rose (1998) also support this, stating that unresolved trauma can shape the ways in which we view certain situations. The circumstances behind this crime may have unearthed some unresolved fear that the British public feels toward men/people in positions of authority, and feel a sense of relation to the crime. Warr (1987) states that women fear crime more than men, as they associate this with a risk of sexual assault. Supporting this, Ferraro (1996) states that women fear sexual crimes whenever they are in public spaces and feel the need to risk assess most situations. Because of Sarah's murder, women are experiencing an added fear; that there are bad apples even in those who are employed to help. It does not matter that this case involved just one officer, the unknown of which officers are good and bad leads to continued fear, anxiety, and collective distrust.

Beyond fear and anxiety, it was identified that some women feared being alone on the streets and felt uncertain of whom they could trust to help them if they were in need. Wattis et al. (2011) found that women link fear and risk to certain places which they have experienced as being dangerous. Moreover, Rodó and Estivill (2016) found that fear can be conditioned where it is experienced. Sarah was walking home from a friend's house while on the phone to her boyfriend when Wayne Couzens abused his powers and used his police badge to falsely arrest her, leading to her rape and murder. With the extreme media coverage surrounding this case, women may now think of Sarah Everard in their future encounters with the police, impacting their trust. Supporting the cultivation hypothesis, which states that those who have been exposed to high levels of crime-related news and media coverage have continued fear of that crime and or victimization (Eschholz et al., 2003). Hirsch (1980) suggests that those victims who deem similar features and characteristics to their self, will invoke a higher fear of the same crime happening to them. Almost half of British women now distrust the police, and 47% of women distrust men after the Sarah Everard case became national news (EVAW, 2021).

Sarah's case was compared to that of the Yorkshire Ripper, who murdered 13 women and attempted to murder seven others in West Yorkshire and Manchester in the 70s and 80s (Kavanagh, 2022). There were many failures within this investigation, with officers often victim-blaming and insufficiently investigating the crime. This led to one of Britain's largest feminism activist protests, where many women and men marched to “reclaim the night” after women were ordered to stay indoors to avoid being murdered (Bindel, 2006). This subsequently led to the Byford enquiry, which revealed many failures in the West Yorkshire investigation (Smith, 2013). In the analysis, many people felt that the police are sexist and misogynistic. In the present day, Sarah's case has made women more fearful that these views are present in police forces. A report released by the IOPC (2022) called Operation Hotton Investigations, investigated misogyny and racism in the metropolitan police and found hundreds of text messages between police officers, with reference to raping and physically abusing women, and laughing and joking about the murders of women. There was also evidence of 17 police officers going to a festival dressed up as known sex offenders and child molesters (IOPC, 2022). When this behavior was brought to the attention of the supervisors, it was dismissed as the officers stated it was “banter” and therefore, got away with it. This report is a public record and adds further concern to the distrust individuals will feel toward the police, when officers have been able to hold such views and get away with it.

Despite these results, some interesting findings were found in relation to the theme, “bad apples in every bunch.” This shows that not all Twitter users perceive the police in a negative light. Around 10 tweets out of 107 viewed police positively, even after hearing about Sarah Everard's murder. Often, most people did not want to judge all officers because of the actions of one man. Twitter posts suggested that there are always bad apples in every bunch, and it would be unfair to treat them all the same because there was one bad apple. However, to contradict this, Baumeister et al. (2001) suggest that a bad apple can spoil a whole barrel. Not only can bad apples spoil the good, but a single bad apple can be strong enough to affect a whole group. This means that despite those individuals viewing the police more positively, the actions of one man are enough to damage the reputation of the entirety of the police. This was enough to make 76% of women feel like policing needs to change in order for them to ever feel safe again following Sarah's murder (EVAWC, 2021).

### Limitations

Like any study, there are limitations within this research project. Firstly, it was ensured that there was no confirmation bias when choosing which tweets to analyze and what to code, however, there is no way to effectively say whether there was 100% no confirmation bias in the selection process or not. The research topic, which was being investigated, is extremely limited in the literature, there is no previous literature surrounding Sarah Everard, and how police perceptions have changed because the case is recent in history, there is no research on it. There was a lack of previous research to build a foundation upon for this study, therefore, a broader literature had to be assessed in order to conduct the research. There are limitations to using social media posts as data, for example, the sample used does not represent the entire population of the British public, as only 1,156 words were analyzed, therefore, we cannot entirely determine to what extent the population views the police force as a whole. It may also not be demographically representative, as Twitter/X's platform user base tends to include younger, urban, and politically engaged individuals, which could limit access to a representative demographic sample. The location of the social media posts was not recorded at the time of data collection; therefore, it is not clear whether the posts were from users from the United Kingdom or users outside of the United Kingdom. For Twitter (now X) specifically, the content often includes sarcasm, memes, and performative posting, which could cause issues interpreting the analog of the posts. The gender of the tweets was not recorded during the data collection process, because it was difficult to assume a person's gender through their social media usernames, and often, people use nicknames and gender neutral terms, rather than their actual names.

### Implications

This research project has provided results which show how the British public's perceptions have dramatically lowered since the murder of Sarah Everard. As a result of her death, confidence in the police has been shattered, and distrust is at an all-time high. More research is needed to fully understand the effect that police officer crimes have on the British public, and how the police force can start to rebuild that trust. Future directions for this research would be to explore police distrust on a nationwide scale, controlling for different demographic variables such as socioeconomic status, education, race, and ethnicity, and also focusing more on gender. It would be recommended to conduct focus groups with the British public to understand these perceptions at face value. A larger-scale study needs to be conducted to fully understand the effects of this issue, and more time needs to be spent to ensure we develop a working pathway to solve the police mistrust in the United Kingdom.

## Conclusion

From this study, we have found that public perceptions have been negatively affected since the Sarah Everard murder and the circumstances surrounding her death. It is hoped that this can offer police forces, the findings from this report to hopefully help them recognize and transform the way in which they can make the public feel safe in the future. How the police can provide all with a safe and trustworthy experience is key to building back that trust. Women and girls, and the whole of the United Kingdom, need to be able to feel safe with the police and know they will not be discriminated against or treated any differently because of who they are.
